# Evaluation of a new self-contained, ambulatory, objective cough monitor

**DOI:** 10.1186/1745-9974-2-7

**Published:** 2006-09-27

**Authors:** Ian M Paul, Kitman Wai, Steven J Jewell, Michele L Shaffer, Vasundara V Varadan

**Affiliations:** 1 Department of Pediatrics, Penn State College of Medicine, Hershey, PA, USA; 2 Department of Health Evaluation Sciences, Penn State College of Medicine, Hershey, PA, USA; 3 Department of Engineering Science and Electrical Engineering, Penn State University, University Park, PA, USA; 4 Department of Electrical Engineering, University of Arkansas, Fayetteville, AR, USA

## Abstract

**Objective and background:**

Objective monitoring of cough may be preferred to subjective reporting of the symptom in clinical and research settings. Therefore, a self-contained, ambulatory cough monitoring system is needed that is non-invasive, usable for children and adults of all ages, inexpensive, and highly accurate with easy to use analysis software.

**Methodology:**

After development of a new device, 15 subjects with frequent coughing were recorded with the novel cough monitor and a simultaneous video recording in order to validate the monitor compared with a gold standard. Two investigators independently analyzed the recordings and counted the number of coughs during the study period from both the cough monitor and the video recording.

**Results:**

When measuring agreement between the two investigators, the sample concordance correlation coefficient for audio counts was 0.998 (*p *< 0.001). In the comparison of video counts, the sample concordance correlation coefficient was 0.997 (*p *< 0.001). For the comparison of investigator 1's video counts to the corresponding audio counts, the sample concordance correlation coefficient was 0.968 (*p *= 0.026). For the comparison of investigator 2's video counts to the corresponding counts, the sample concordance correlation coefficient was 0.973 (*p *= 0.015).

**Conclusion:**

We have developed and piloted a new, valid, and reproducible method of objectively recording and analyzing cough. This device appears to be useful for subjects of any age and in clinical and research settings.

## Background

Cough is one of the most bothersome symptoms of illness, and is the most common cause of outpatient acute care visits in the United States each year [1]. The causes of cough are varied and multi-factorial, ranging from simple upper respiratory infections to pneumonia to chronic conditions such as asthma and emphysema. Despite the extremely common nature of this symptom and its variability based on etiology, cough is typically assessed only subjectively in clinical and research settings. Even the most expensive clinical trials related to diseases such as asthma assess cough subjectively with diary cards where patients report cough frequency and severity [2]. This is concerning since subjective reporting of cough has been shown to be unreliable and inconsistently accurate particularly for nighttime symptoms and for the reporting of symptoms in children [3-12].

As such, we developed a self-contained, ambulatory cough monitoring system that was designed to be non-invasive, usable for children and adults of all ages, inexpensive, and highly accurate for the detection of cough with easy to use software for data analysis. Upon its completion, we aimed to pilot its ability to accurately record cough frequency and validate its accuracy in quantitating cough by comparing the auditory recordings of the device with simultaneously performed video recordings serving as the gold-standard.

## Materials and methods

### Cough monitor

**Figure 1:**
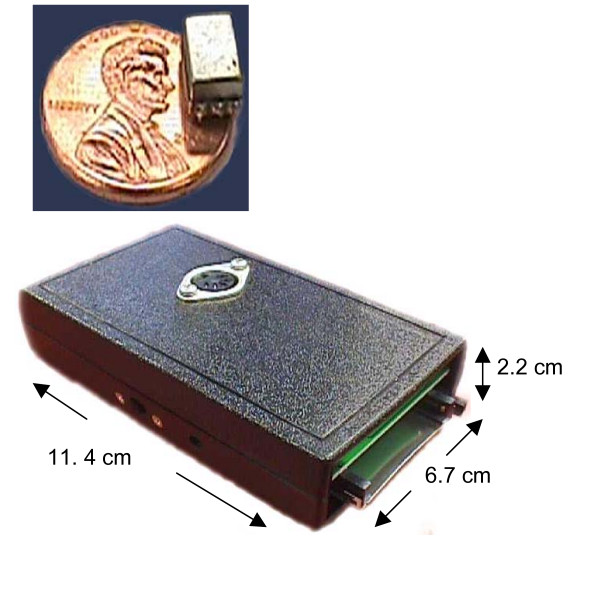
The cough monitoring system: a) accelerometer and b) electronic package.

The self-contained monitor consists of several components: 1) an accelerometer, 2) an electronic package (dimensions 11.4 cm × 6.7 cm × 2.2 cm, weight 171 grams), 3) a cable connecting the accelerometer to the electronic package, and 4) a CompactFlash memory card. The accelerometer chosen was the BU-1771 (Figure 1a; Knowles Electronics Co., Itasca, Illinois, USA), and it is attached to the skin at each subject's suprasternal notch as has been done previously using a bioclusive transparent dressing [13]. The accelerometer measures vibration at this location, and transmits output data through a cable to an electronic package (Figure 1b) that is typically worn on the belt or in a pocket. There the signal is amplified and a microprocessor performs an analog to digital conversion before storing the data on the CompactFlash memory card (Lexar Media, Inc., Freemont, CA, USA). The monitor is capable of storing 24 hours of data on a 1 GB CompactFlash Card, and is powered by a 9 V battery that is contained within the electronic package.

Attachment at the suprasternal notch is advantageous for numerous reasons. First, because it is below the larynx, any speech that causes vibrations is unintelligible on the audio recording. This maintains the privacy for the subject that may be recorded for extended periods in an ambulatory setting. Next, it eliminates any interference from swallowing. Third, it is a relatively comfortable location that does not interfere with typical daily activities. Lastly, this location and its method of placement eliminate the problem of movement artifact or distance from an externally located microphone.

### Software

**Figure 2:**
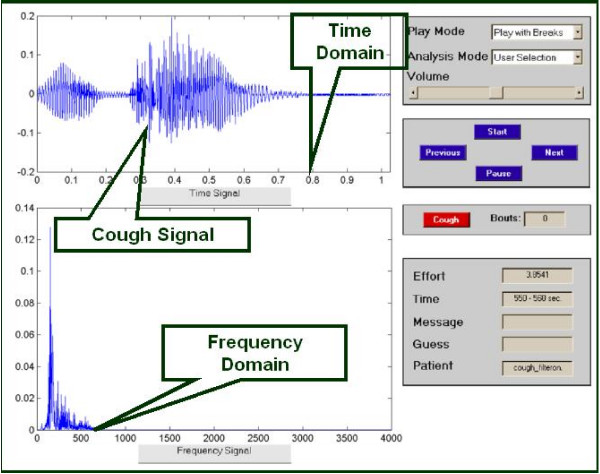
Data analysis software: Graphical user interface (GUI).

**Figure 3:**
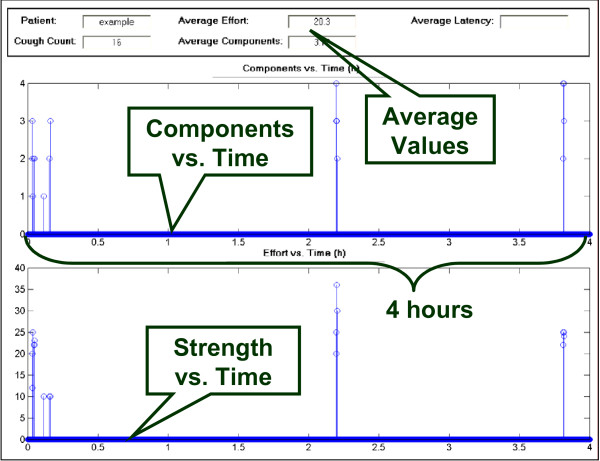
Data analysis software: Post-analysis data summary plot.

The analysis software presents the stored data from the CompactFlash card in a user-friendly manner that allows the user to verify whether a recorded signal represents a cough or not. Importantly, the software can run the stored recording continuously or eliminate silent periods where no signal occurs. The latter feature greatly reduces the time required for analysis. Using a program developed through Matlab^® ^(The MathWorks, Natick, MA, USA), a graphical user interface (GUI) enables the user to analyze the cough recordings (Figure 2). The user determines whether a signal is a cough or not based on its visual features in time and frequency domains including the visualized slope and typical pattern as described previously [14] as well as its sound, which is played from a '.wav' audio file. The combination of visual and audio features allows for easy distinction between other noises such as speech, laughter, or throat clearing. Also, for each cough detected, the intensity of the cough is also calculated. Finally, the GUI generates a post-analysis plot of the data for a summary of the recording (Figure 3).

### Participants and recordings

15 subjects with very frequent coughing when evaluated subjectively, or their legal guardians, consented to be recorded with the cough monitor and simultaneous video recording for a period ranging from 15 to 60 minutes. Though the monitor can we worn for much longer periods of time, given the time consuming nature of reviewing longer period of video recording, short periods were selected for this study. Subjects were recorded in the outpatient clinic, hospital, and home environments between November 2004 and February 2005. Subjects were recorded in the home, outpatient, inpatient, and outdoor settings. The Human Subjects Protection Office of the Penn State College of Medicine approved the study.

### Cough definitions and recording analysis

As has been done previously, a "cough bout" was defined as a one-second period of time where the subject was coughing [13,15]. During each cough bout, one or more "cough components" could occur. A cough component was defined as individual bursts of air that the patient emits during a cough. Each cough component begins with the first audible phase of a cough.

In this study, only the cough components were analyzed and each component will be referred to as a "cough" for the remainder of this manuscript. Two investigators independently analyzed the recordings and counted the number of coughs during the study period from both the cough monitor and the video recording. The two investigators were in the same room at the time of the recording analyses, but were blinded to each other's interpretation. Interpretation of what constituted a cough on the video recordings remained subject to investigator discretion. One investigator had no prior experience in cough research, but the second investigator had prior experience with objective cough recordings.

### Statistical analysis for validation

Using the video recordings as a gold-standard,[16] a sample size calculation indicated that with a total of 23 subjects, a one-sided 95% confidence interval for the concordance correlation coefficient would have a lower limit of 0.90, assuming the true concordance correlation coefficient was 0.95 with variance 1.00. An interim analysis was planned to determine if the sample size required expansion or reduction because the number of coughs per recording was difficult to predict a priori. The concordance correlation coefficient is a reproducibility index that captures precision and accuracy [17]. Any value larger than 0 indicates agreement with 1 indicating perfect agreement. We established a priori that an acceptable level of agreement, as measured by the concordance correlation coefficient, is 0.90. Several parameters were compared in this analysis, including agreement between video counts and audio counts, agreement of audio counts between investigators, and agreement of video counts between investigators. The null hypothesis is the level of agreement is less than or equal to 0.90. Our hypothesis was that level of agreement would be greater than 0.90. We conducted a one-sided hypothesis test at the 0.05 level of significance as well as computed a 95% lower confidence bound. If the *p*-value was below 0.05, we would reject the null hypothesis and conclude we had adequate agreement. Similarly, if the lower confidence bound lay above 0.90, we would conclude that we had adequate agreement.

## Results

15 subjects aged 2 weeks to 84 years with cough were enrolled and completed the study (Table 1). A variety of diagnoses was identified as the cause of each subject's cough. Though the recordings ranged between 15 and 60 minutes, all subjects demonstrated relatively frequent coughing during the study periods that allowed for a comparison of cough counts by video and monitor recordings between methods and between investigators.

**Table 1:** Patient characteristics and cough recording results (video and monitor) as determined by two investigators

**Age**	**Diagnosis**	**Recording Duration**	**Investigator 1**	**Investigator 2**	**Investigator 1**	**Investigator 2**
		minutes	Video count	Video count	Monitor count	Monitor count
60 yrs	Pneumonia	30	43	41	50	51
20 mos	Bronchiolitis	60	53	50	43	40
2 yrs	Asthma/pneumonia	30	45	45	48	47
55 yrs	COPD	30	40	42	34	37
13 yrs	Pneumonia	30	37	36	39	39
2 wks	Pertussis	30	62	63	63	63
23 yrs	Allergic Rhinitis	30	29	32	28	29
6 yrs	Upper Respiratory infection	15	74	73	74	74
43 yrs	Upper respiratory infection	15	80	81	81	82
45 yrs	Upper respiratory infection	30	14	14	16	16
79 yrs	Pneumonia	30	17	19	15	15
16 yrs	Upper respiratory infection	20	49	49	40	40
49 yrs	Asthma	30	14	13	14	17
7 yrs	Upper respiratory infection	30	30	28	25	25
28 yrs	Upper respiratory infection	30	69	68	64	64

When measuring agreement between the two investigators, the sample concordance correlation coefficient for audio counts from the new device was 0.998 with a 95% lower confidence bound of 0.994 (*p *< 0.001). In the comparison of video counts, the sample concordance correlation coefficient was 0.997 with 95% lower confidence bound of 0.991 (*p *< 0.001). There was good agreement between the two investigators' audio counts. There was also good agreement between the two investigators' video counts, and experience with objective cough recordings did not impact the findings.

Next, the agreement between video counts and audio counts were calculated for both investigators. For the comparison of investigator 1's video counts to the corresponding audio counts, the sample concordance correlation coefficient was 0.968 with a 95% lower confidence bound of 0.918 (*p *= 0.026). For the comparison of investigator 2's video counts to the corresponding counts, the sample concordance correlation coefficient was 0.973 with a 95% lower confidence bound of 0.930 (*p *= 0.015). The agreement level was slightly higher for the more experienced counter; however, this difference was not statistically significant (*p *= 0.990).

## Discussion

To overcome the deficiencies of subjective reporting of cough, numerous attempts have been made to develop objective cough monitoring devices. Methods have ranged from very simple devices consisting of a tape recorder placed in a room with a patient to complex devices capable of measuring multiple physiologic parameters including cough [6,13-15,18-29]. Most if not all of these systems have some limitation that makes them difficult to use in a subject's natural environment with a routine level of activity or does not protect the privacy of their vocal conversations during the recording period.

The results of this study describe a newly developed method of objectively recording and analyzing cough. Though the recordings were for a short durations, the method appears valid and reproducible. Though these findings are limited by the fact that we did not match each video recorded cough to the device recorded cough, the device we describe combines several patient-friendly features since it is a non-invasive, self-contained, and ambulatory device. This distinguishes it from other devices since it does not appear to interfere in any way with routine, daily activities. We also have demonstrated its potential utility in subjects that are very young as well as those that are senior citizens with a variety of medical diagnoses serving as the etiology of their coughing. The software that accompanies the device is user-friendly and produces easily understandable analyses.

Because subjective reporting of cough has been shown to be unreliable, clinical trials that assess the common symptom of cough should consider objective assessments. The device we describe here can also be used in the clinical setting to evaluate the frequency of a patient's cough. With further work and the help of acoustics experts, the visual and audio analysis components could be explored as a diagnostic tool to determine the etiology of a cough. It also could potentially be adapted to evaluate other pulmonary sounds such as snoring, stridor, or wheezing.

## Competing interests

The author(s) declare that they have no competing interests.
